# Central Sensitivity Is Associated with Poor Recovery of Pain: Prediction, Cluster, and Decision Tree Analyses

**DOI:** 10.1155/2020/8844219

**Published:** 2020-10-30

**Authors:** Hayato Shigetoh, Masayuki Koga, Yoichi Tanaka, Shu Morioka

**Affiliations:** ^1^Department of Neurorehabilitation, Graduate School of Health Sciences, Kio University, Nara, Japan; ^2^Miura Internal Medicine Michiko Pediatrics Clinic, Kagawa, Japan; ^3^Neuro Rehabilitation Research Center, Kio University, Nara, Japan

## Abstract

The process of pain recovery varies and can include the recovery, maintenance, or worsening of symptoms. Many cases of patients with pain show a tendency of recovering as predicted; however, some do not. The characteristics of cases that do not fit the prediction of pain recovery remain unclear. We performed cluster and decision tree analyses to reveal the characteristics in cases that do not fit the prediction of pain recovery. A total of 43 patients with musculoskeletal pain (nonoperative: 22 patients, operative: 13 patients) and central pain (brain disease: 5 patients, spinal cord disease: 3 patients) were included in this longitudinal study. Central sensitivity syndrome (CSS) outcome measures (Central Sensitisation Inventory), pain intensity-related outcome measures (Short-Form McGill Pain Questionnaire-2 (SFMPQ-2)), and cognitive-emotional outcome measures (Hospital Anxiety and Depression Scale and Pain Catastrophising Scale-4) of all patients were assessed at baseline and after 1-2 months. Regression analysis was used to calculate pain recovery prediction values. A hierarchical cluster analysis based on the predicted change of SFMPQ-2 and the observed change of SFMPQ-2 was used to extract subgroups that fit and those that do not fit pain recovery prediction. To extract the characteristics of subgroups that do not fit the prediction of pain recovery, a decision tree analysis was performed. The level of significance was set at 5%. In the results of cluster analysis, patients were classified into three subgroups. Cluster 1 was characterised by worse pain intensity from baseline, cluster 2 by pain, having recovered less and mildly than the predicted value, and Cluster 3 by a marked recovery of pain. In the results of the decision tree analysis, the CSI change was extracted as an indicator related to the classification of all clusters. Our findings suggest that the poor improvement of CSS is characteristic in cases that do not fit the prediction of pain recovery.

## 1. Introduction

Pain is not only a symptom of musculoskeletal disorders [1–3] but also a symptom of many other diseases such as central nervous system disorders [4, 5], and the severity of pain varies from mild to severe [6–8]. Many cases tend to recover [8, 9]; however, the amount of improvement is varied. In addition, it has been reported that pain intensity can be maintained or can worsen [6–11]. Many longitudinal studies have predicted postintervention pain severity, often from the initial pain score [12–14]. Several longitudinal studies have reported central sensitivity syndrome (CSS) [15, 16], catastrophic thinking [17], anxiety [18], and depression [14, 18, 19] in the initial state as predictors of postintervention pain. However, these predictors are predictors of postintervention pain severity, and the relationships between cognitive-emotional factors, CSS, and predictors of pain recovery have not been clarified. It is important to capture the change value rather than the postintervention value because a recovery process fundamentally implies a change in the state rather than just an endpoint. In addition, a relationship between the pain intensity change and the patient's global impression of improvement has been reported [10]. In the recent years, pain recovery prediction models have been developed, and the accuracy of the agreement between predicted and observed pain recovery has been examined. In pain recovery prediction, observed pain recovery has been less or more than predicted pain recovery [9, 20, 21]. However, it is unclear what factors affect the failure to recover as predicted.

Severe pain is associated with hypersensitivity, which is induced by central sensitisation (CS) [22–25]. The International Association for the Study of Pain defines CS as the “increased responsiveness of nociceptive neurons in the central nervous system to their normal or subthreshold afferent input” [26], and CS is one of the factors which exacerbate pain. On the other hand, CSS is a symptom associated with CS in the absence of organic abnormalities [27]. The central sensitisation inventory (CSI) has recently been used as a comprehensive screening instrument for CSS [28–30]. Several studies revealed that pain intensity was associated not only with psychological factors [31, 32] but also with the CSI score [24, 29, 31, 33]. Our previous study also reported that the CSI score is associated with pain intensity [34]. However, as our study was a cross-sectional study, we could not reveal the relationships between cognitive-emotional factors, CSS, and pain intensity in the pain recovery process. Thus, it is unclear how cognitive-emotional factors and CSS are related to pain intensity change.

Furthermore, the characteristics of cognitive-emotional factors and CSS in cases that do not fit the prediction of pain recovery have been unclear. Identifying these characteristics may lead to tailor-made interventions. The aim of the present study was (1) to create a pain recovery prediction model based on cases of pain that recovered and to extract cases that fit and those that do not fit pain recovery prediction and (2) to reveal the characteristics in cases that do not fit the prediction of pain recovery. We hypothesised that there are subgroups that do not fit pain recovery prediction and have little recovery or worsening of pain. We also expected that poor initial cognitive-emotional factors and CSS and/or poor improvement of cognitive-emotional factors and CSS may be extracted as characteristics in cases that do not fit the prediction of pain recovery.

## 2. Patients and Methods

### 2.1. Study Design

**Figure 1:**
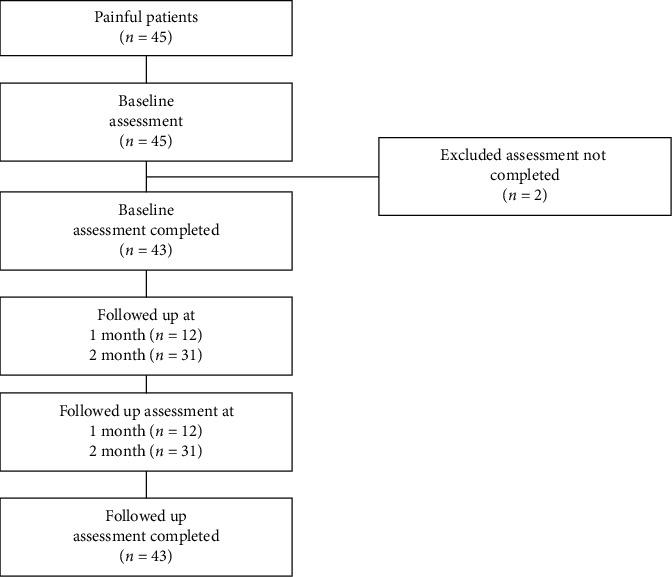
Progression of participants through the trial including losses to follow-up.

Forty-three patients with pain who received physical therapy were recruited from inpatient hospital admissions (*n* = 29) and outpatient orthopaedic clinics (*n* = 14). We wanted to investigate the effect of CS on pain without identifying diseases susceptible to CS, so we did not limit the diagnosis of a disease and painful area. Also, we did not limit the pain duration when investigating the various pain conditions. Exclusion criteria were patients with a diagnosis of dementia or significantly higher brain dysfunction and difficulty in answering the questionnaire. All patients were assessed at baseline and had started physical therapy from before baseline. Then, all patients were assessed again at 2 months follow-up after baseline. If patients did not receive treatment for 2 months after baseline due to discharge from the hospital, we assessed them at the 1-month follow-up after baseline. The follow-up period for each patient was 12 patients at 1 month and 31 patients at 2 months (Figure 1). The study protocol conformed to the Declaration of Helsinki. The participants provided written informed consent before the study began. This study was approved by the ethics committee of Kio University Health Sciences Graduate School (approval no. H30-06).

### 2.2. Evaluation of Patient Characteristics by the Questionnaire

The following characteristics were assessed for each patient: demographic data (age and gender), diagnosis, pain duration, pain intensity (Short-Form McGill Pain Questionnaire-2), CSSs (Central Sensitisation Inventory-9 (CSI-9)), cognitive-emotional factors (Hospital Anxiety and Depression Scale (HADS), and Pain Catastrophising Scale-4 (PCS-4)). The diagnosis was classified as nonoperative and operative for musculoskeletal diseases, while central nervous system diseases were classified as brain diseases or spinal cord diseases.

The SFMPQ-2 was used to assess pain intensity [35] and included items that assessed 22 qualities of pain and the intensity of each quality on an 11-point numerical rating scale. The total score was calculated from the sum of the 22 items. Higher scores indicate more severe pain.

The CSI-9 was used to assess health-related symptoms that are common to CSS [36]. CSI-9 is a shorter version of a 25-item CSI and contains 9 items. Higher scores indicate more severe CSS.

The HADS was used to assess anxiety and depression as psychological factors. The HADS contains 14 items and 2 subscales [37]. The two subscales independently assessed anxiety (HADS-A) and depression (HADS-D). Higher scores indicate more severe anxiety and depression.

The PCS-4 was used to assess catastrophic thinking. PCS-4 is a shorter version of a 13-item PCS and contains 4 items [38]. Higher scores indicate more severe catastrophic thinking.

### 2.3. Statistical Analyses

G^∗^Power software version 3.1.9.7. was used to calculate the effect size of the Mann–Whitney *U*-test. We applied Mann–Whitney to calculate the sample size because our hypothesis was that patients would divide into, at least, two groups. An a-priori power analysis for a Mann–Whitney *U*-test was conducted in to determine a sufficient sample size using an alpha of 0.05, a power of 0.80, and a large effect size (*d* = 0.80), as well as one tail.

In this study, the analysis was performed in stages. (1) To create a predictive equation for pain recovery, pain recoverers were selected. Pain change (∆SFMPQ-2) was calculated using the SFMPQ-2 follow-up–SFMPQ-2 baseline. Negative values of ∆SFMPQ-2 indicated recovery, and positive values of ∆SFMPQ-2 indicated worsening. The patients of negative values of ∆SFMPQ-2 were defined as pain recoverers. (2) According to the recovery prediction equation, the slope (*m*) plus *y*-intercept (*b*) model relating recovery (*R*) to initial impairment (*I*) is *R* = *mI* + *b* [39], and regression analysis from the selected pain recoverers was used to create a prediction equation for pain recovery. (3) Using the pain recovery prediction equation, we calculated the predicted pain recovery values (∆SFMPQ-2 predicted) and observed pain recovery values (∆SFMPQ-2 observed). (4) A hierarchical cluster analysis based on ∆SFMPQ-2 predicted and ∆SFMPQ-2 observed was used to extract subgroups that fit and those that do not fit pain recovery predictions. A hierarchical cluster analysis was performed using Ward's clustering method with Euclidean distances. The optimal number of clusters was determined by examining the agglomeration coefficients and analysis of the dendrogram. (5) To compare variables (demographics, pain, cognitive-emotional factors, and CSS) between subgroups, Fisher's exact test was performed for categorical variables. Also, one-way analysis of variance (ANOVA), the Kruskal–Wallis test, and multiple comparisons using Holm's adjustment method to optimize protection against Type 1 error were performed for continuous variables. (6) A preliminary Spearman's correlation matrix was performed to understand the associations between all assessed questionnaire variables. In particular, we focused on the relationship between pain change (∆SFMPQ-2), cognitive-emotional factor changes (∆HADS-A, ∆HADS-D, and ∆PCS-4), and CSS change (∆CSI-9). “∆” indicates the follow-up score minus the initial score. A Pearson correlation analysis was used for the association between CSS change (∆CSI-9) and cognitive-emotional factor changes (∆HADS-A, ∆HADS-D, and ∆PCS-4) according to the normal distribution. As baseline pain intensity could be considered important for pain prognosis, the Partial Correlation Coefficient (PCC) was used as a measure of association between the change of pain intensity and change of pain-related factors. Briefly, PCC is a measure of the association for two continuous variables controlled for the effect of baseline pain intensity that is considered confounders. (7) To extract the characteristics of subgroups that do not fit the prediction of pain recovery, a decision tree analysis with CART (classification and regression trees) was performed [40]. The focus of the decision tree was to facilitate the diagnostic decision between clusters. The baseline value and change value of all questionnaire variables (the CSI-9 baseline, ∆CSI-9, HADS-A baseline, ∆HADS-A, the HADS-D baseline, ∆HADS-D, PCS-4 baseline, and ∆PCS-4) were included in the tree model. The classification and regression tree (CART) methodology with the Gini index rule, one of the decision tree analyses, was used for developing CPR and assessing the accuracy. The pruning rule in the CART was used the minimum cross-validation error with 1 standard error rule. We evaluated the performance of the decision tree classifier by Leave-one-out cross-validation (42 patients were selected as training data, and 1 patient was selected as test data). Statistical analyses were performed with *R*, ver. 3.6.1. The level of significance was set at *p* < 0.05.

## 3. Results

### 3.1. Pain Recovery Prediction Model Using Regression Analysis

Of the 43 patients, 25 recovered from pain. Data from 25 patients were used to create a pain recovery prediction equation. The pain recovery prediction equation was ∆SFMPQ-2 = −0.52^∗^SFMPQ-2 baseline − 3.34. The adjusted coefficient of determination (*R*^2′^) was 0.56. The coefficient of determination was used to measure the goodness of fit between actual and predicted data. In general, if the coefficient of determination is 0.5≦, the accuracy of the regression analysis is considered to be good [41].

### 3.2. Cluster Analysis and Comparison between Clusters

The cluster analysis based on ∆SFMPQ-2 predicted and ∆SFMPQ-2 was classified into three subgroups. Cluster 1 (*n* = 10) was characterised by pain that recovered to less than the predicted value and pain for which the intensity became worse. Cluster 2 (*n* = 21) was characterised by pain that recovered to less than the predicted value and pain that recovered mildly. Cluster 3 (*n* = 12) was characterised by pain that recovered just as predicted and pain that recovered markedly. Figure 1 shows the scatter diagram using the ΔSFMPQ-2 predicted and ΔSFMPQ-2 observed values categorised by clusters.

Patient characteristics by clusters are summarised in Table 1. Gender was significantly different between clusters. Age, pain duration, and diagnosis classification did not show any significant difference between clusters.

**Figure 2:**
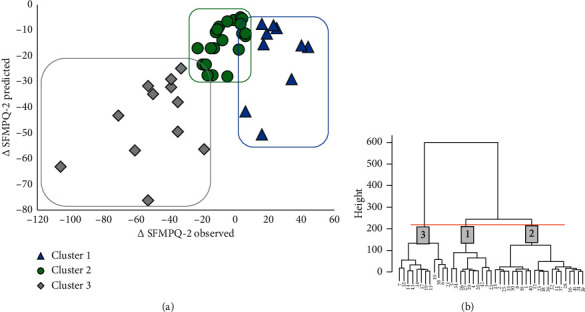
(a) The scatter plot of the ∆SFMPQ-2 observed score and ∆SFMPQ-2 predicted score. Triangle indicates cluster 1. Circle indicates cluster 2. Rhombus indicates cluster 3. (b) Dendrogram of cluster analysis. Abbreviations: SFMPQ-2, Short-Form McGill Pain Questionnaire-2; ∆, follow-up score minus the initial score.

In the SFMPQ-2 baseline, cluster 3 was significantly higher than cluster 1 and cluster 2. The results of the trend test showed a trend toward higher SFMPQ-2 baseline values from cluster 1 to cluster 3. In the SFMPQ-2 follow-up, it was significantly higher in cluster 1 than in clusters 2 and 3. The results of the trend test showed a trend toward lower SFMPQ-2 follow-up values from cluster 1 to cluster 3. In the ∆SFMPQ-2 group, cluster 1 showed significantly more recovery than cluster 2 and cluster 3, and cluster 2 showed significantly more recovery than cluster 3. The results of the trend test showed a trend toward ∆SFMPQ-2 observed more recovery from cluster 1 to cluster 3. In the ∆SFMPQ-2 predicted, cluster 3 showed significantly more recovery than clusters 2 and 3. The results of the trend test showed that a trend toward ∆SFMPQ-2 predicted more recovery from cluster 1 to cluster 3. In the ∆SFMPQ-2 observed and predicted errors (∆SFMPQ-2 observed-predicted), cluster 1 showed worse pain recovery than clusters 2 and cluster 3, and cluster 2 showed worse pain recovery than cluster 3 (Figure 2).

In the CSI-9 baseline, cluster 3 was significantly higher than cluster 2. The results of the trend test did not show a significant trend. In the CSI-9 follow-up, there were no significant differences between the clusters. The results of the trend test did not show a significant trend. In ∆CSI-9, cluster 3 showed significantly more improvement than cluster 2. The results of the trend test showed a trend toward ∆CSI-9 more improvement from cluster 1 to cluster 3.

**Figure 3:**
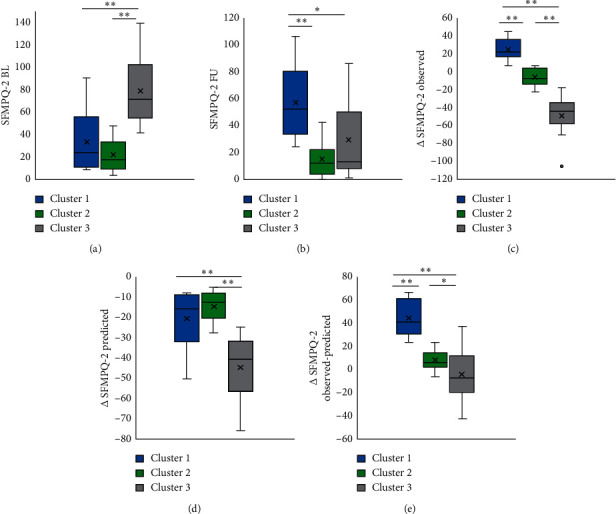
The difference of SFMPQ-2 between clusters. (a) The difference of SFMPQ-2 BL between clusters. (b) The difference of SFMPQ-2 FU between clusters. (c) The difference of ∆SFMPQ observed between clusters. (d) The difference of ∆SFMPQ-2 predicted between clusters. (e) The difference of ∆SFMPQ-2 observed-predicted between clusters. ^*∗∗*^:*p* < 0.01. ^*∗*^: *p* < 0.05. Abbreviations: SFMPQ-2, Short-Form McGill Pain Questionnaire-2; BL, baseline; FU, follow-up; ∆, follow-up score minus the initial score.

All the HADS-A baseline, HADS-A follow-up, and ∆HADS-A had no significant differences between the clusters. The results of the trend test did not show a significant trend in the HADS-A baseline, HADS-A follow-up, and ∆HADS-A. Similarly, there was no significant difference between clusters in all the HADS-D baseline, HADS-D follow-up, and ∆HADS-D. The results of the trend test showed no significant trend in the HADS-D baseline, HADS-D follow-up, and ∆HADS-D (Figure 3).

In the PCS-4 baseline, there were no significant differences between clusters. The results of the trend test did not show a significant trend. In the PCS-4 follow-up, there were no significant differences between clusters. The results of the trend test did not show a significant trend. In the ∆PCS-4, there were no significant differences between clusters. The results of the trend test showed a trend toward ∆PCS-4 more improvement from cluster 1 to cluster 3.

### 3.3. Correlation Analysis

**Figure 4:**
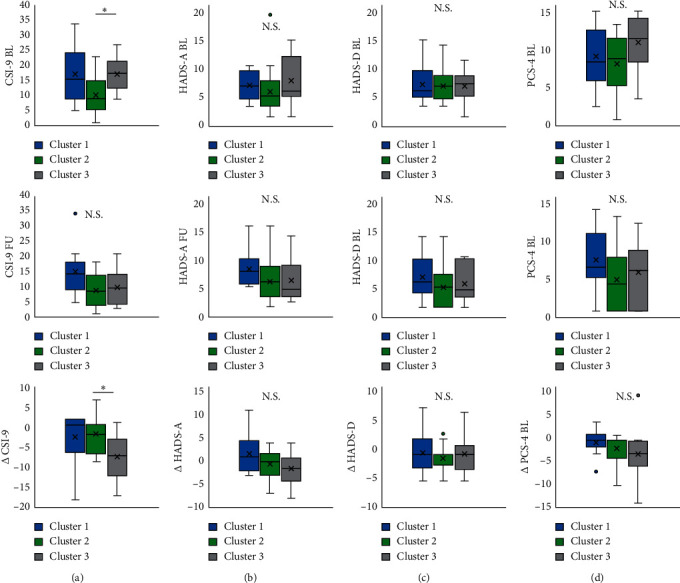
The difference of CSI-9, HADS-A, HADS-D, and PCS-4 between clusters. (a) The difference of CSI-9 between clusters. (b) The difference of HADS-A between clusters. (c) The difference of HADS-D between clusters. (d) The difference of PCS-4 between clusters. ^*∗*^: *p* < 0.05. Abbreviations: CSI-9, Central Sensitisation Inventory-9; HADS-A, Hospital Anxiety and Depression Scale-Anxiety; HADS-D, Hospital Anxiety and Depression Scale-Depression; PCS-4, Pain Catastrophising Scale-4; BL, baseline; FU, follow-up; ∆, follow-up score minus the initial score.

Spearman's correlation matrix is summarised in Table 2. The correlation coefficients and scatter plot between pain change and CSS change/cognitive-emotional factors change are summarised in Figure 4. ∆SFMPQ-2 showed a significant positive correlation with the ∆CSI-9 (*ρ* = 0.42, 95% CI: 0.13, 0.64, *p* < 0.01) and ∆PCS-4 (*ρ* = 0.43, 95% CI: 0.15, 0.65, *p* < 0.01). On the other hand, ∆SFMPQ-2 showed no significant correlation with ∆HADS-A (*ρ* = 0.22, 95% CI: −0.09, 0.49, *p*=0.16) and ∆HADS-D (*ρ* = 0.16, 95% CI: −0.15, −0.44, *p*=0.32). The PCC is a measure of the relationship between pain change and CSS change/cognitive-emotional factors change controlled for the effect of baseline pain intensity that is considered confounders. The PCC is added in Figure 4. In the partial correlation analysis, ∆SFMPQ-2 showed a significant positive correlation with the ∆CSI-9 (*p* < 0.05), ∆HADS-D (*p* < 0.05), and ∆PCS-4 (*p* < 0.01). On the other hand, ∆SFMPQ-2 showed no significant correlation with ∆HADS-A (*p*=0.26).

**Figure 5:**
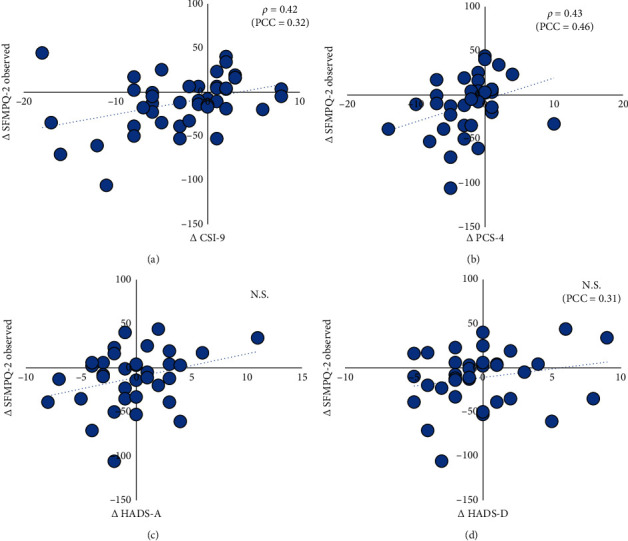
The correlation analysis and scatter plot of ∆SFMPQ-2 and ∆CSI-9, ∆HADS-A, ∆HADS-D, and ∆PCS-4. (a) The correlation analysis and scatter plot of ∆SFMPQ-2 and ∆CSI-9. (b) The correlation analysis and scatter plot of ∆SFMPQ-2 and ∆PCS-4. (c) The correlation analysis and scatter plot of ∆SFMPQ-2 and ∆ HADS-A. (d) The correlation analysis and scatter plot of ∆SFMPQ-2 and ∆HADS-D. Abbreviations: SFMPQ-2, Short-Form McGill Pain Questionnaire-2; CSI-9, Central Sensitisation Inventory-9; HADS-A, Hospital Anxiety and Depression Scale-Anxiety; HADS-D, Hospital Anxiety and Depression Scale-Depression; PCS-4, Pain Catastrophising Scale-4; PCC, Partial Correlation Coefficients; ∆, follow-up score minus the initial score.

**Figure 6:**
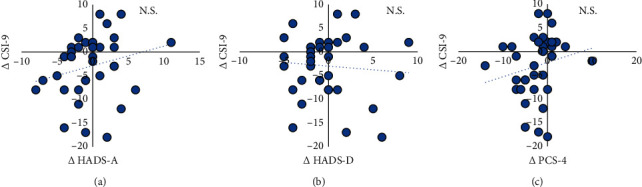
The correlation analysis and scatter plot of ∆CSI-9 and ∆HADS-A, ∆HADS-D, and ∆PCS-4. (a) The correlation analysis and scatter plot of ∆CSI-9 and ∆ HADS-A. (b) The correlation analysis and scatter plot of ∆CSI-9 and ∆HADS-D. (c) The correlation analysis and scatter plot of ∆CSI-9 and ∆PCS-4. Abbreviations: CSI-9, Central Sensitisation Inventory-9; HADS-A, Hospital Anxiety and Depression Scale-Anxiety; HADS-D, Hospital Anxiety and Depression Scale-Depression; PCS-4, Pain Catastrophising Scale-4; ∆, follow-up score minus the initial score.

The correlation coefficients and scatter plot between CSS change and changes in cognitive-emotional factors are summarised in Figure 5. ∆CSI-9 showed no significant correlation with the ∆PCS-4 (*r* = 0.20, 95% CI: −0.11, 0.47, *p*=0.21), ∆HADS-A (*r* = 0.23, 95% CI: −0.08, 0.50, *p*=0.14), and ∆HADS-D (*r* = −0.08, 95% CI: −0.37, −0.23, *p*=0.62) (Figure 6).

### 3.4. Decision Tree Analysis

**Figure 7:**
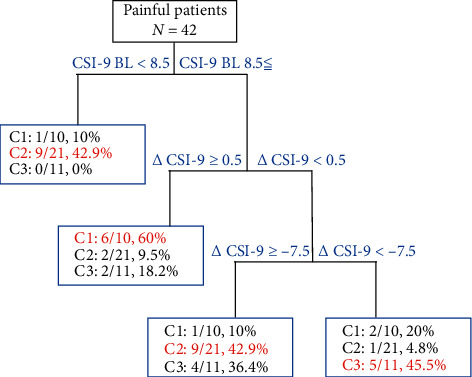
Decision tree analysis for cluster classification. The first predictive factor was CSI-9 BL < 8.5 or CSI-9 BL 8.5≦. The second predictive factor was ∆CSI-9 ≥ 0.5 or ∆CSI-9 < 0.5. The third predictive factor was ∆CSI-9 ≥ −7.5 or ∆CSI-9 < −7.5. Each square box shows the percentage of cluster affiliation when each predictor is met. Abbreviations: CSI-9, Central Sensitisation Inventory-9; BL, baseline; ∆, follow-up score minus the initial score; C, cluster.

The final tree model identified using CART analysis and training data (*n* = 42) are shown in Figure 7. The final tree model consisted of the splits according to CSI-9 baseline (CSI-9 baseline <8.5 or CSI-9 baseline 8.5≦) and ∆CSI-9 (∆CSI-9 ≧ 0.5 or ∆CSI-9 < 0.5, and ∆CSI-9 ≧ −7.5 or ∆CSI-9 < −7.5). As indicators for selecting cluster 1, CSI-9 BL 8.5≦ and ∆CSI-9 0.5≦ were extracted, and 60% of total cluster 1 was selected. As indicators for selecting cluster 2, (1) CSI-9 baseline <8.5 and (2) CSI-9 baseline 8.5≦ and −7.5 ≦ ∆CSI-9 < 0.5≦ were extracted, respectively. In addition, (1) 42.9 % and (2) 42.9% of total cluster 2 were selected. As indicators for selecting cluster 3, CSI-9 baseline 8.5≦ and ∆CSI-9 < −7.5 were extracted, and 45.5% of total cluster 3 was selected. The accuracy of final tree model was 69.0% in the training data (*n* = 42). Also, the mean accuracy of final tree model was 61.1% in the test data. Thus, this tree model was 7.9% overfitted.

## 4. Discussion

We created the pain recovery prediction equation and used cluster analysis based on ∆SFMPQ-2 predicted and ∆SFMPQ-2 observed to investigate the characteristics of subgroups that do not fit the prediction of pain recovery. Patients were classified into three subgroups. Cluster 1 was characterised by worse pain intensity. Cluster 2 was characterised by pain that recovered less than the predicted value and pain that recovered mildly. Cluster 3 was characterised by a marked recovery of pain. The results showed a trend toward higher severity of pain at baseline from cluster 1 to cluster 3 and a trend toward lower severity of pain at follow-up from cluster 1 to cluster 3. Focusing on CSS, the CSS change showed significantly more improvement in cluster 3 than in cluster 2 and a trend toward CSS with more improvement from cluster 1 to cluster 3. In addition, focusing on cognitive-emotional factors, only catastrophic thinking showed a trend toward more improvement from cluster 1 to cluster 3. In the results of the correlation analysis, the amount of pain intensity change showed a significant positive correlation with CSS change and catastrophic thinking change. In addition, the CSS change showed no significant correlation with catastrophic thinking change. In the results of the decision tree analysis, the severity of CSS at baseline and CSS change were extracted as indicators for selecting cluster classification. Interestingly, all clusters were divided into clusters by CSS change. However, this decision tree model was a bit overfitted. Therefore, the present study showed that pain recovery was associated with improvement of CSS and catastrophic thinking, and poor improvement of CSS was extracted as characteristics in cases that did not fit the prediction of pain recovery.

The present study is the first to demonstrate how CSS and cognitive-emotional factors are associated with not fitting of pain recovery prediction. Previous studies have reported that even if the initial pain is severe or moderate, pain recovers [6, 7]. Similar results were shown in the present study, and the pain intensity of cluster 3 was high at baseline. In addition, in the present study, CSI-9 baseline score and CSI-9 change were selected as variables associated with cluster classification, but cognitive-emotional factors were not selected. The CSI score at baseline has also been reported as a variable associated with the prognostic value of pain and disability [15, 42]. However, the CSI-9 scores of cluster 1 and cluster 3, which were conflicting in pain recovery in the present study, were similar. On the other hand, the CSI-9 change showed a trend toward more improvement from cluster 1 to cluster 3. The results of the decision tree analysis showed that a worsening CSI-9 had a high probability of being selected for cluster 1 (the pain was worse), a mild CSI-9 improvement had a high probability of being selected for cluster 2 (the pain was less recovered than predicted), and a high CSI-9 improvement had a high probability of being selected for cluster 3 (the pain was greatly recovered). Thus, it is suggested that poor improvement of the CSS is a characteristic of cases that do not fit the prediction of pain recovery.

Another feature of this study was to investigate the relationship between pain, CSS, and cognitive-emotional factors, focusing on the amount of change. The results of the present study showed that the change in pain intensity correlated with the change in CSS and change in catastrophic thinking, whereas the change in pain intensity did not correlate with the change in anxiety and depression. In our previous study, CSS and catastrophic thinking were also associated with pain intensity [34], and the results of the amount of change in the present study showed a similar trend. In addition, previous cross-sectional studies have reported a correlation between CSI and PCS [43, 44]. However, the change in CSS was not correlated with the change in catastrophic thinking. This may indicate that CSS and catastrophic thinking are associated with pain exacerbation through different mechanisms. This mechanism may have a physiological explanation. Several mechanisms, including CS, peripheral sensitisation, cognitive-emotional sensitisation, and interpersonal sensitisation, have been reported as mechanisms of pain exacerbation [45]. These results suggested that CSS changes were influenced by CS and led to worsening pain, and catastrophic thinking changes were influenced by cognitive-emotional sensitisation and led to worsening pain. In this study, we were able to extract CSS changes as a characteristic of cases that did not fit the prediction of pain improvement. CSI has also been reported to be associated with central nervous systems involved in pain, such as brain-derived neurotrophic factor (BDNF) [46] and gamma-aminobutyric acid (GABA) [47]. In addition, BDNF and GABA are associated with pain intensity. Hence, the change in CSS may affect the activity of the central nervous system, such as BDNF and GABA, which may affect poor pain recovery.

Our findings suggest that differences in CSS improvement can lead to nonfit pain recovery prediction. CSI scores have been reported to vary independently of CSI severity [33, 48]. Therefore, it may be necessary to consider not only the CSI score at baseline but also the improvement of CSS. Focusing on the treatment, patient education based on pain neuroscience [33, 49] and exercise therapy [24, 50] have been reported to be effective in improving CSS, along with improving pain. Moreover, CSS has also been associated with psychological stress symptoms [31] and the central nervous system [46, 47]. There may be a need for comprehensive pain management focused on the central nervous system.

This study had several limitations. (1) We could not determine the mechanisms underlying the relationship between pain intensity, CSS, and psychological factors because this study did not measure neurotransmitter levels. (2) As the timing of the reassessment was not controlled, it may have had an impact on the results by different periods of intervention. (3) The course and number of sessions of physical therapy for each patient could not be controlled. The course of drug therapy was also unclear. Differences in each patient's physiotherapy and drug therapy content may have affected the outcome after follow-up. However, all patients received weekly physical therapy, and the purpose of this study was not to determine the intervention's effectiveness. This study focused on the association between pain and pain-related factor change scores. Therefore, the purpose of this study was achieved. (4) The sex ratio of the patients could not be adjusted. Gender differences may have affected the outcome. (5) Other factors such as body perception disturbance [51] have been reported as factors affecting pain. However, this study did not examine other factors. Revealing the relationships between pain change and other factors such as body perception disturbance in future research may lead to tailor-made treatment planning with a clearer picture of the factors to focus on. (6) With reference to a previous study [50–52], the optimal number of clusters was determined by examining the agglomeration coefficients and analysis of the dendrogram, taking into account the sample size of each cluster. However, this method also includes analyst bias in selecting the number of clusters, so it may have been necessary to choose the number of clusters using the cross-validation method. (7) The sample size of this study is not large. The results of this study might be based on a small heterogeneous sample. Also, this small sample size may have led to multiple comparisons and overfitting of the decision tree. Also, the accuracy of the decision tree was not very high. Therefore, it may lead to misclassification of the clusters. (8) This study did not reveal complex causal relationships between pain and pain-related factors, such as the change of pain led to the change of pain-related factors or change of pain-related factors that affected the change of pain. Future studies may reveal detailed causal relationships using future statistical analysis, such as cross-lag correlation analysis. (9) This study included elements of nociceptive pain, neuropathic pain, and nociplastic pain because of the variety of diseases included in the study. It was difficult to determine which type of pain was the specific result of this study. Future research needs to examine the details of the interventions and what factors need to be improved to improve the type of pain.

## 5. Conclusions

To our knowledge, this is the first study to investigate how CSS and cognitive-emotional factors are associated with pain that does not fit recovery prediction. Our findings suggest that poor improvement of the CSS is a characteristic of cases that do not fit to the prediction of pain recovery. Our findings could help reduce pain intensity in patients with pain.

## Figures and Tables

**Table 1:** Characteristics of each variable in each cluster.

Variable	Total	Cluster 1 (*n* = 10)	Cluster 2 (*n* = 21)	Cluster 3 (*n* = 12)	*p* value	Trend test^¶^
Age (years)	72.2 ± 12.9	75.0 ± 8.8	72.6 ± 9.3	69.2 ± 19.2	*p*=0.58^*∗*^	—
Gender (female)	10 (33)	2 (8)	8 (13)	0 (12)	*p* < 0.05^§^	—
Pain duration (month)	11.8 ± 30.2	20.2 ± 34.7	8.2 ± 25.1	11.2 ± 32.8	*p*=0.66^*∗*^	—
Disease classification	MSK-NO : 22	MSK-NO : 7	MSK-NO : 10	MSK-NO : 5	*p*=0.21^§^	—
	MSK-O : 13	MSK-O : 2	MSK-O : 5	MSK-O : 6		
	CND-B : 5	CND-B : 0	CND-B : 5	CND-B : 0		
	CND-S : 3	CND-S : 1	CND-S : 1	CND-S : 1		
SFMPQ-2 BL	40.0 ± 33.2	32.7 ± 27.1	21.4 ± 14.2	78.8 ± 28.9	*p* < 0.01^*∗*^	*p* < 0.01
SFMPQ-2 FU	28.4 ± 27.1	56.7 ± 25.9	14.5 ± 11.5	29.3 ± 28.7	*p* < 0.01^*∗*^	*p* < 0.05
∆SFMPQ-2 observed	−11.6 ± 30.1	24.0 ± 11.3	−6.9 ± 9.1	−49.5 ± 21.7	*p* < 0.01^*#*^	*p* < 0.01
∆SFMPQ-2 predicted	−24.3 ± 17.4	−20.5 ± 14.2	−14.5 ± 7.4	−44.6 ± 15.1	*p* < 0.01^*∗*^	*p* < 0.01
∆SFMPQ-2 observed-predicted	12.7 ± 23.3	44.4 ± 15.2	7.7 ± 7.7	−4.9 ± 21.2	*p* < 0.01^#^	*p* < 0.01
CSI-9 BL	13.8 ± 7.4	17.3 ± 9.0	10.2 ± 5.8	17.3 ± 5.5	*p* < 0.01^#^	*p*=0.44
CSI-9 FU	11.1 ± 6.9	15.7 ± 8.3	9.3 ± 5.5	10.4 ± 6.1	*p*=0.15^*∗*^	*p*=0.17
∆CSI-9	−2.7 ± 6.0	−1.6 ± 6.5	−0.9 ± 4.5	−6.8 ± 6.0	*p* < 0.05^*∗*^	*p* < 0.01
HADS-A BL	5.8 ± 4.2	6.1 ± 2.7	4.9 ± 4.3	7.1 ± 4.7	*p*=0.43^*∗*^	*p*=0.85
HADS-A FU	5.6 ± 4.1	7.6 ± 3.5	4.5 ± 3.8	5.8 ± 4.2	*p*=0.13^*∗*^	*p*=0.25
∆HADS-A	−0.2 ± 3.3	1.5 ± 4.0	−0.4 ± 2.6	−1.3 ± 3.2	*p*=0.37^#^	*p*=0.15
HADS-D BL	6.1 ± 3.2	6.3 ± 3.7	6.0 ± 2.9	6.0 ± 3.1	*p*=0.98^#^	*p*=0.89
HADS-D FU	5.9 ± 3.5	6.7 ± 3.6	5.3 ± 3.4	6.2 ± 3.4	*p*=0.65^#^	*p*=0.85
∆HADS-D	−0.2 ± 3.1	0.4 ± 4.1	−0.6 ± 2.0	0.2 ± 3.5	*p*=0.65^*∗*^	*p*=0.84
PCS-4 BL	9.4 ± 4.3	9.3 ± 4.2	8.2 ± 4.0	11.4 ± 4.1	*p*=0.12^#^	*p*=0.18
PCS-4 FU	7.1 ± 4.4	8.6 ± 3.4	5.9 ± 4.3	8.0 ± 4.6	*p*=0.25^#^	*p*=0.96
∆PCS-4	−2.3 ± 3.8	−0.7 ± 2.8	−2.3 ± 2.9	−3.4 ± 5.3	*p*=0.06^#^	*p* < 0.01

Values are numbers for categorical variables and mean and SD for continuous variables unless otherwise indicated. Abbreviations: SFMPQ-2, Short-Form McGill Pain Questionnaire-2; CSI-9, Central Sensitisation Inventory-9; HADS-A, Hospital Anxiety and Depression Scale-Anxiety; HADS-D, Hospital Anxiety and Depression Scale-Depression; PCS-4, Pain Catastrophising Scale-4; BL, baseline; FU, follow-up; ∆, follow-up score minus the initial score; MSK-NO, musculoskeletal-nonoperative; MSK-O, musculoskeletal-operative; CND-B, central nerve disease-brain; CND-S, central nerve disease-spinal cord. ^*∗*^: assessed by one-way analysis of variance. #: assessed by the Kruskal–Wallis test; ^§^: assessed by Fisher's exact test. Assessed by the Jonckheere–Terpstra trend test.

**Table 2:** A preliminary Spearman's correlation matrix between all assessed questionnaire variables.

	SFMPQ-2 BL	SFMPQ-2 FU	ΔSFMPQ-2	CSI-9 BL	CSI-9 FU	ΔCSI-9	HADS-A BL	HADS-A FU	ΔHADS-A	HADS-D BL	HADS-D FU	ΔHADS-D	PCS-4 BL	PCS-4 FU	ΔPCS-4
SFMPQ-2 BL	1.00														
SFMPQ-2 FU	0.42^*∗∗*^	1.00													
ΔSFMPQ-2	−0.60^*∗∗*^	0.36^*∗*^	1												
CSI-9 BL	0.59	0.49^*∗∗*^	−0.12	1											
CSI-9 FU	0.30^*∗*^	0.61^*∗∗*^	0.23	0.56^*∗∗*^	1										
ΔCSI-9	−0.28	0.15	0.42^*∗∗*^	−0.45^*∗∗*^	0.41^*∗∗*^	1									
HADS-A BL	0.40^*∗∗*^	0.37^*∗*^	−0.07	0.48^*∗∗*^	0.23	−0.29	1								
HADS-A FU	0.29	0.53^*∗∗*^	0.14	0.51^*∗∗*^	0.47^*∗∗*^	−0.07	0.60^*∗∗*^	1							
ΔHADS-A	−0.13	0.14	0.22	−0.04	0.22	0.28	−0.52^*∗∗*^	0.30^*∗*^	1						
HADS-D BL	0.18	−0.01	−0.18	−0.02	−0.04	0.03	0.15	0.19	−0.03	1					
HADS-D FU	0.15	0.44^*∗∗*^	0.03	0.40^*∗∗*^	0.37^*∗*^	−0.02	0.35^*∗*^	0.59^*∗∗*^	0.22	0.55^*∗∗*^	1				
ΔHADS-D	0.30	0.43^*∗∗*^	0.16	0.44^*∗∗*^	0.47^*∗∗*^	0.04	0.22	0.40^*∗∗*^	0.24	−0.38^*∗*^	0.49^*∗∗*^	1			
PCS-4 BL	0.47^*∗∗*^	0.28	−0.22	0.37^*∗*^	0.36^*∗*^	−0.13	0.26	0.43^*∗∗*^	0.15	0.29	0.50^*∗∗*^	0.20	1		
PCS-4 FU	0.46	0.61^*∗∗*^	0.02	0.55^*∗∗*^	0.68^*∗∗*^	0.03	0.40^*∗*^	0.52^*∗∗*^	0.16	−0.18	0.45^*∗∗*^	0.38^*∗*^	0.60^*∗∗*^	1	
ΔPCS-4	−0.11^*∗∗*^	0.44^*∗∗*^	0.43^*∗∗*^	0.12	0.41^*∗∗*^	0.33^*∗*^	0.05	0.16	0.10	0.05	−0.003	0.18	−0.35^*∗*^	0.43^*∗∗*^	1

Abbreviations: SFMPQ-2, Short-Form McGill Pain Questionnaire-2; CSI-9, Central Sensitisation Inventory-9; HADS-A, Hospital Anxiety and Depression Scale-Anxiety; HADS-D, Hospital Anxiety and Depression Scale-Depression; PCS-4, Pain Catastrophising Scale-4; BL, baseline; FU, follow-up; ∆, follow-up score minus the initial score. ^*∗∗*^:*p* < 0.01. ^*∗*^: *p* < 0.05.

## Data Availability

The data used to support the findings of this study are included within the article.
